# His-tag based supramolecular biopolymerization

**DOI:** 10.1038/s41598-024-78647-1

**Published:** 2024-11-16

**Authors:** Mitra Lal, Ellina Kesselman, Ellen Wachtel, Olga Kleinerman, Yoav Peleg, Shira Albeck, Koushik Majhi, Mordechai Sheves, Guy Patchornik

**Affiliations:** 1 Department of Chemical Sciences, Ariel University, 70400 Ariel, Israel; 2 Department of Chemical Engineering, Technion-Israel Institute of Technology, 3200003 Haifa, Israel; 3 Faculty of Chemistry, Weizmann Institute of Science, 7610001 Rehovot, Israel; 4 Structural Proteomics Unit (SPU), Department of Life Sciences Core Facilities (LSCF), Weizmann Institute of Science, 7610001 Rehovot, Israel; 5 Department of Molecular Chemistry and Materials Science, Weizmann Institute of Science, 7610001 Rehovot, Israel

**Keywords:** Biochemistry, Biological techniques

## Abstract

The term supramolecular polymer has been applied to polymeric materials in which the individual units, i.e., building blocks—are bound to each other via noncovalent interactions, including electrostatic or hydrogen bonding, as well as metal–ligand conjugation. The building blocks are generally low molecular weight amphiphiles. Methods for preparing biopolymers based on non-toxic, metal–ligand conjugation have been little studied; however, they offer significant potential for tuning the response of biologically relevant macromolecules. In this communication, we characterize the assembly and morphology of supramolecular biopolymers in which the building blocks are low- or medium-molecular weight globular proteins—ubiquitin and Cas9-interacting via metal–ligand conjugation. In each case, the protein gene was expressed in cell culture with the addition of hexa-His/linkers at both the N and C termini. Divalent cations investigated were Zn^2+^ and Ni^2+^. We observe in cryo-TEM imaging an absolute requirement for divalent cations for the formation of supramolecular biopolymers. In the presence of Ni^2+^, 1D assembled fibers are predominant, while with Zn^2+^, the more frequently detected structures are sheet-like. We use gel electrophoresis and CD spectroscopy to monitor possible secondary and tertiary structural changes in the protein building blocks during conjugation.

**Supplementary Information:**

The online version contains supplementary material available at 10.1038/s41598-024-78647-1.

## Introduction

The term supramolecular polymer has been applied to polymeric materials in which the monomeric units i.e., the building blocks—are bound to each other via noncovalent interactions, including electrostatic or hydrogen bonding, as well as metal-ligand conjugation^1^ and references cited therein. The building blocks are generally low molecular weight amphiphiles such as: amphiphilic peptides^2^, oligo-peptides modified with aromatic groups^3^, synthetic small molecules bound to urea and aromatic groups^4^. Polymeric precursors terminated with metal-binding groups, including histidine or catechol, have been observed to form hydrogels in the presence of metal ions such as Zn^2+^, Fe^2+^ and Fe^3+^^5–7^. Although methods for preparing biopolymers based on non-toxic metal-ligand conjugation have been little studied^1^ they offer significant potential for controlling and tuning the response of biologically relevant macromolecules.

**Fig. 1:**
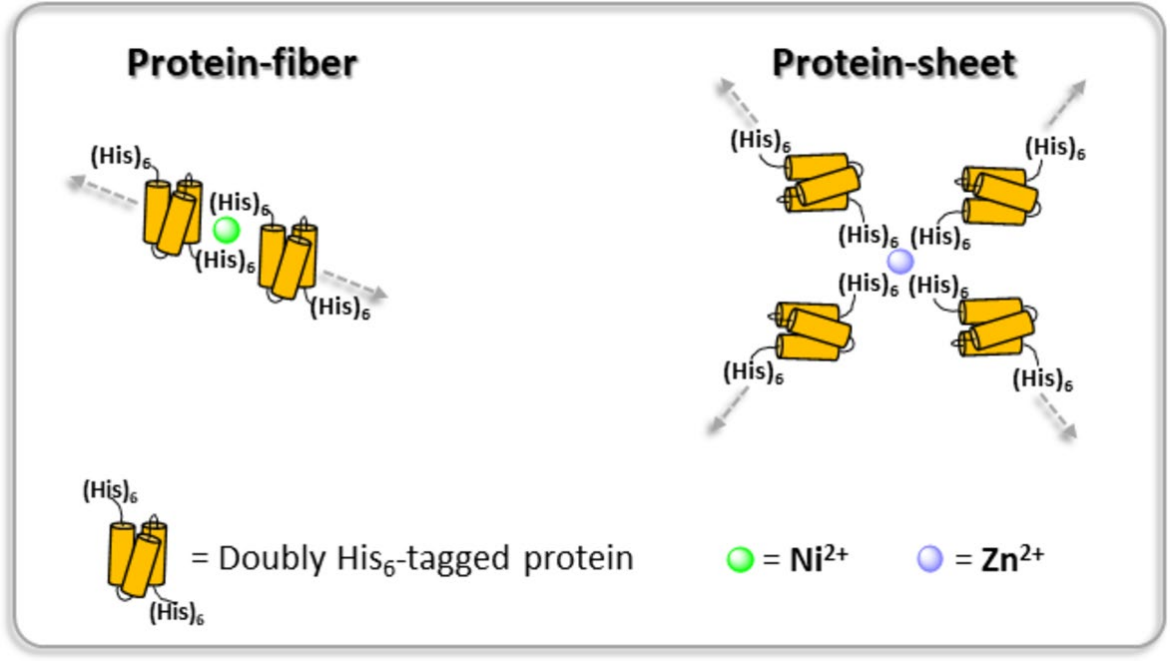
Cartoon of hexa-His ([His_6_]) ligand–metal conjugation with the potential of promoting self-assembly of protein fibers or sheets as macromolecular biopolymers. [His_6_]-tags are added to expression genes in *E. coli* BL21(DE3) competent cells at both the N- and C-terminal ends of the proteins. Dimensions are not to scale.

In this communication, we characterize the assembly and morphology of supramolecular biopolymers in which the building blocks are low- or medium-molecular weight globular proteins interacting *via* metal-ligand conjugation (Fig. 1). The proteins we have chosen are ubiquitin (Ub; 8.6 KDa) and Cas9 (160 KDa). Ubiquitin plays a vital role in cell homeostasis by labelling molecules for degradation^8,9^. Cas9 is an enzyme associated with the Clustered Regularly Interspaced Short Palindromic Repeats (CRISPR) adaptive immune system in Streptococcus pyogenes (sp) and has been productively used in genome engineering to introduce site-directed double-strand breaks in DNA^10^. The ligand we have employed in both cases is the His_6_-tag ([His_6_]), well known as an effective aid in column chromatography protein purification^11–13^. The ubiquitin gene (2–76) with an N-terminal hexa-His-tag/linker (MGSSHHHHHHSAGSAGSAG) and a hexa-His-tag/linker at the C-terminus (SAGSAGSAGHHHHHH) was expressed in cell culture, as was SpCas9 with the same N-terminal hexa-His-tag/linker and a different hexa-His-tag/linker at the C-terminus (SRADPKKKRKVAAALEHHHHHH). Divalent cations investigated were Zn^2+^ and Ni^2+^. Dissociation constants of the divalent cations with His_6_-tags vary over 20 orders of magnitude (K_d_ (Ni^2+^) = 0.88 µM; K_d_ (Zn^2+^) = 0.047 µM)^14^. We use native- and SDS-PAGE electrophoresis of the [His_6_]_2_ tagged building blocks; monitor maintenance of native secondary structure by circular dichroism (CD) spectroscopy; demonstrate the absolute requirement for divalent cations; and characterize the supramolecular biopolymeric structures by cryo-TEM imaging in vitreous ice. We observe in cryo-TEM images that in the presence of Ni^2+^ ions, both proteins predominantly form fibers, while with Zn^2+^, the detected structures are predominantly sheet- or membrane-like (Fig. 1). We attribute the difference in morphology, at least in part, to the difference in binding constants of the divalent cations to the hexa-His tags.

## Results

### Macromolecular UB-[His_6_]_2_ polymerization promoted by ligand-divalent cation interactions

**Fig. 2:**
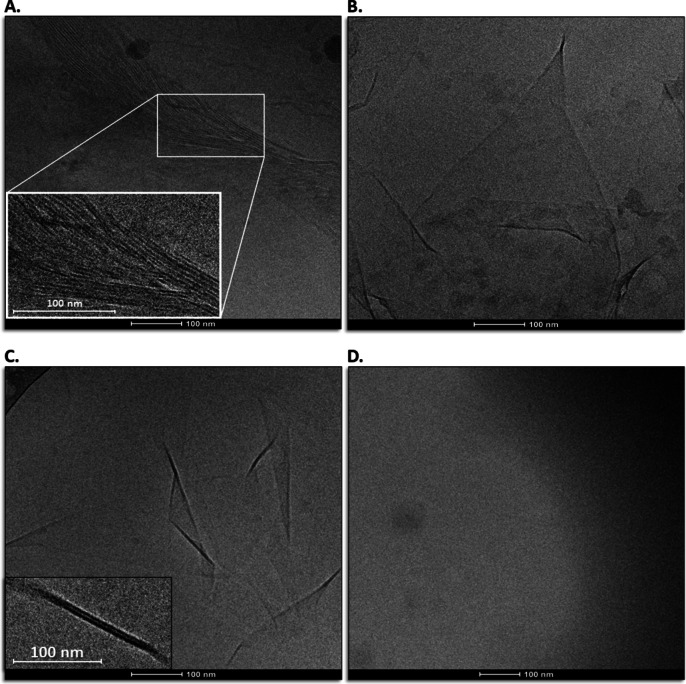
(**A**) Cryo-TEM imaging of fibrous protein in vitreous ice obtained approx. 10 min after addition of 10 µM NiCl_2_ to 0.05 mg/mL UB-[His_6_]_2_ in 30 mM Tris, pH 7.5, 25 °C. (**B**) Cryo-TEM image of 0.05 mg/mL UB-[His_6_]_2_ following 10 min incubation in 30 mM Tris pH 7.5 at 25 °C with the addition of 10 mM ZnCl_2_. (**C**) Cryo-TEM image of 3.8 mg/mL UB-[His_6_]_2_ following overnight incubation in 30 mM Tris pH 7.5 at 19 °C with the addition of 100 mM ZnCl_2_. Folded sheets/membranes are observed. (**D**) Cryo-TEM image of 3.8 mg/mL UB-[His_6_]_2_ in in the absence of Ni^2+^ or Zn^2+^ with overnight incubation at 19 °C.

With cryo-TEM imaging, we observe the formation of ubiquitin (UB) fibers following 10 min room temperature incubation of UB-[His_6_]_2_ (0.05 mg/mL, ~ 6 µM) with 10 µM Ni^2+^ ions in Tris-HCl buffer, pH 7.5 (Fig. 2A). The small molar excess of Ni^2+^ over UB-[His_6_]_2_, suggests tight binding of Ni^2+^ to the His_6_-tags which is consistent with the known binding affinity of recombinant His_6_-tagged proteins to Ni-NTA resins (i.e., K_d_=1 µM^15^to 10 µM^16^). The binding affinity of two His_6_-tags to Ni^2+^ with K_d_= 0.88 µM has also been determined^14^. The measured width of a fiber is estimated to be ~ 4 ± 1 nm (Fig. 2A). Since the effective diameter of a UB molecule is ~ 2.5 nm^17^, this suggests that each fiber may be built from some form of UB dimers. Fiber lengths can exceed > 500 nm and, in some cases, 6–8 fibers were observed to be aligned in parallel arrays (Fig. 2A).

While most forms of ubiquitin (UB) oligomerization in vivo are due to N-terminal/C-terminal covalent peptide linkage, Liu et al., have reported^18^ that free UB is also capable of dimerizing noncovalently in solution with monomer-dimer equilibrium constant K_d_= 4.9 ± 0.3 mM. The minimum concentration at which dimers appear to form is 0.2 mM, during which two ubiquitin molecules bury a relatively large, solvent-accessible area located within β-sheet regions. Interaction of a metal ion with the histidine end-group requires deprotonation of both of the imidazole groups. The addition of divalent metal ions at or near a stoichiometric ratio resulted in significant stabilization of the deprotonated state of histidine end-groups as seen by titration curves^5^. The propensity for two His_6_-tags to dimerize in the presence of Zn^2+^ is ~ 20-fold greater than with Ni^2+^ and is consistent with measured metal dissociation constants from His_6_-tags, i.e., K_d_ [Zn^2+^] = 0.047 µM vs. K_d_ [Ni^2+^] = 0.88 µM^14^. Furthermore, the crystal structure of hexaimidazole dichloride tetrahydrate reveals that Zn^2+^ can participate in either tetrahedral or octahedral coordination with the free nitrogens of the imidazole moiety^19^.

Polymer assembly was repeated with Zn^2+^ under conditions similar to those used for Ni^2+^. Sheets, rather than fiber morphology, were observed (Fig. 2B; Supplementary Information, Fig. S1). With increased protein concentration, as well as with modified time and temperature of incubation, (Fig. 2C), i.e., overnight incubation at 19 °C with Zn^2+^, imaging again showed sheet-like morphology. Measurement of the approximate width of the folded sheet tips gave 3–3.5 nm. The absolute requirement for divalent cations is demonstrated in Fig. 2D, where ordered, one- or two- dimensional assembly is not observed. An additional control was performed: UB-[His_6_]_2_ was replaced by UB-[His_6_]_1_, but with the same protocol followed as for Fig. 2B. The absence of ordered, protein assemblies demonstrated the mandatory participation of both C- and N-terminal His_6_-tags (Supplementary Information, Figure S2).

### Macromolecular Cas9-[His_6_]_2_ polymerization promoted by ligand-divalent cation interaction: cryo-TEM imaging

**Fig. 3:**
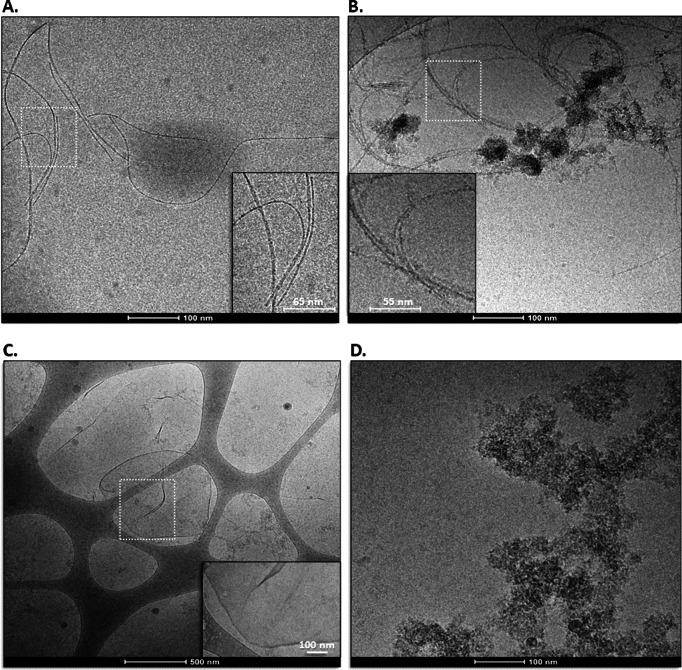
(**A**,** B**) Cryo-TEM imaging of fibrous protein in vitreous ice obtained approx. 10 min after addition of 10 µM NiCl_2_ to 0.05 mg/mL Cas9-[His_6_]_2_ in 30 mM Tris, pH 7.5, 25 °C. (**C**) Cryo-TEM image of 0.05 mg/mL Cas9-[His_6_]_2_ following 10 min incubation in 30 mM Tris pH 7.5 at 25 °C with the addition of 10 mM ZnCl_2_. Folded sheets/membranes are observed. (**D**) Cryo-TEM image of concentrated (3.8 mg/mL) Cas9-[His_6_]_2_ in the absence of Ni^2+^ or Zn^2+^, incubated overnight in 30 mM Tris pH 7.5 at 19 °C. Organized supramolecular structures are not observed, rather disorganized aggregates.

When UB-[His_6_]_2_ was replaced by Cas9-[His_6_]_2_, fibers > 500 nm long were observed in the presence of Ni^2+^ (Fig. 3A, B). Fiber width was estimated to be approximately 7 nm. Since native, apo-Cas9 has a crescent, bi-lobed, conformationally flexible structure in solution^20^ it is difficult to predict what will be its orientation in the fibrous assembly in vitreous ice. Under similar conditions, but in the presence of Zn^2+^, approximately 500 nm x 500 nm two-dimensional sheets were generated (Fig. 3C) with a folding pattern similar to that observed for UB-[His_6_]_2_. The approximate width of a folded tip was estimated to be 6.4 nm. In the absence of divalent cations, ordered assembly of Cas9-[His_6_]_2_ into macromolecular polymers was not observed (Fig. 3D).

### Circular dichroism (CD) spectroscopy

Circular dichroism (CD) spectroscopy is an excellent tool for rapid determination of the secondary structure and folding properties of proteins. The most widely used application of protein CD spectra is the determination of whether a protein displays its native fold, or if a mutation affects its conformation or stability. In addition, it can be used to study protein interactions. We have therefore studied the interactions of Cas9-[His_6_]_2_ and UB-[His_6_]_2_ with Ni^2+^ or Zn^2+^ by CD spectroscopy.

**Fig. 4:**
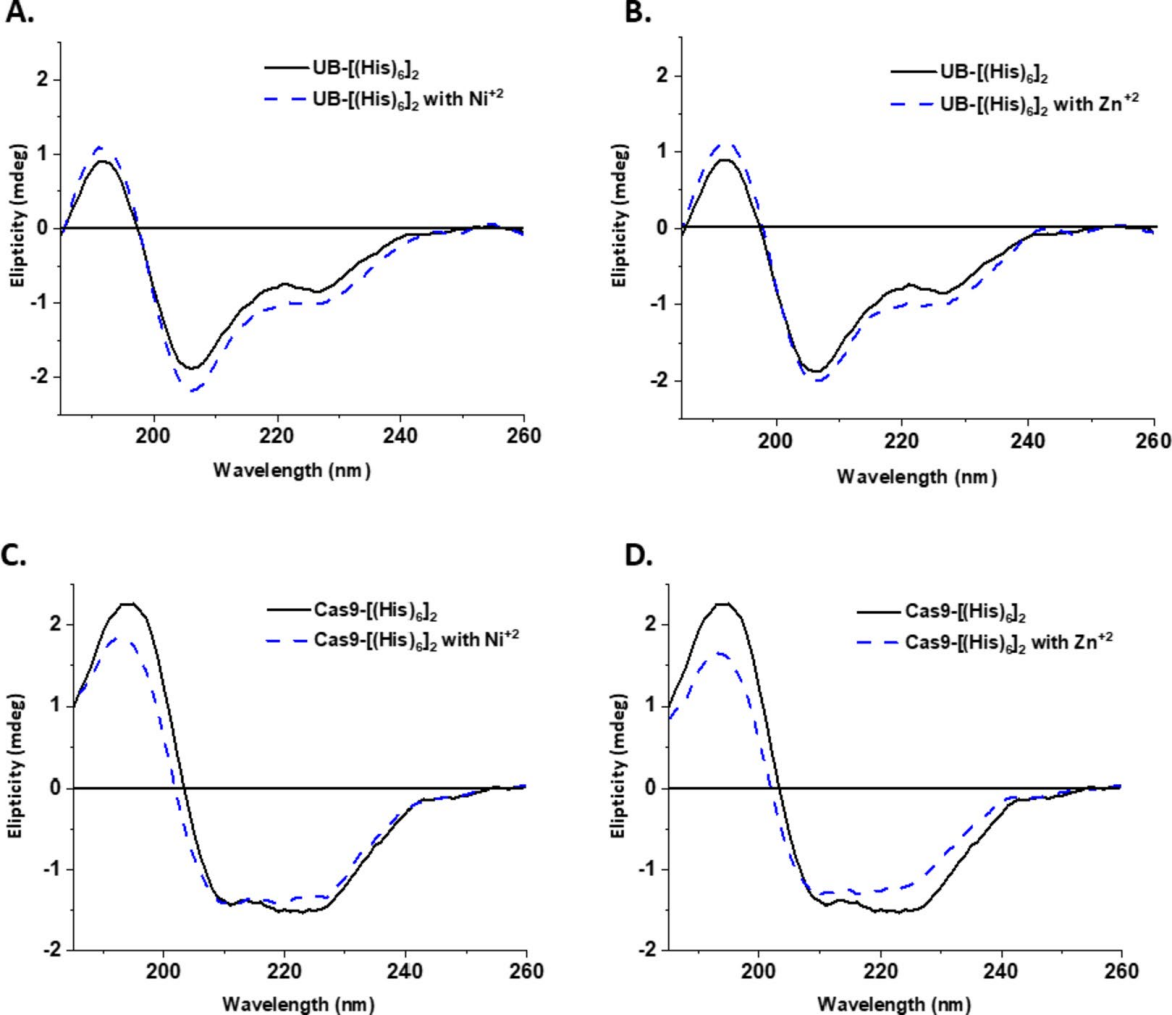
Far UV circular dichroism (CD) spectra of 0.05 mg/mL doubly His-tagged UB (top row **A**,** B**) or Cas9 (bottom row **C**,** D**) with 10 µM NiCl_2_ (left hand panels **- -**) or ZnCl_2_ (right hand panels **- -**) in DDW at 25 °C. Spectra measured in the absence of metal ions (**---**) were used as control. Background has been subtracted.

Chiral peptide bonds of a protein give rise to characteristic CD spectral features at wavelengths < 240 nm that are sensitive to the secondary structure of the protein fold – alpha-helix, beta-sheet, etc. The experimental CD spectrum of native Cas9 protein displays a negative ellipticity peak/positive ellipticity peak pattern, with two negative bands of nearly the same intensity, observed at 221 and 209 nm, accompanied by a positive peak at 196 nm^21^. Recombinant Cas9-[His_6_]_2_, dissolved in DDW, with or without conjugation *via* Zn^2+^ or Ni^2+^, show negative ellipticity peaks at ~ 224 nm and ~ 210 nm, and a positive ellipticity peak at ~ 194 nm (Fig. 4). Native mono-ubiquitin in PBS, pH 7, 37 °C displays three prominent ellipticity peaks: the first between 190 and 200 nm (positive), the second at ~ 205 nm (negative) along with a third, ~ 30% weaker, negative peak at ~ 225 nm^22^. Recombinant UB-[His_6_]_2_, with or without conjugation via Ni^2+^ or Zn^2+^, shows a positive ellipticity peak at 192 nm and negative peaks at 207 and 228 nm. Since the pKa of the imidazole side chain in histidine is ~ 6 while the pH of DDW varies between 5.5 and 6.9, we may assume that the pH during the CD measurements was sufficiently high to prevent protonation of the imidazole rings in the His-tags and consequently to promote chelation of the added metal, thereby leading to organized polymerization. Any changes that could be detected in the CD spectra of the recombinant [His_6_]-tagged proteins after 10 min incubation at 25 °C, when compared to the native spectra in the literature^21,22^ were considered to be insignificant (Fig. 4).

### Monitoring changes in electrophoretic mobility: SDS-PAGE and Native-PAGE

**Fig. 5:**
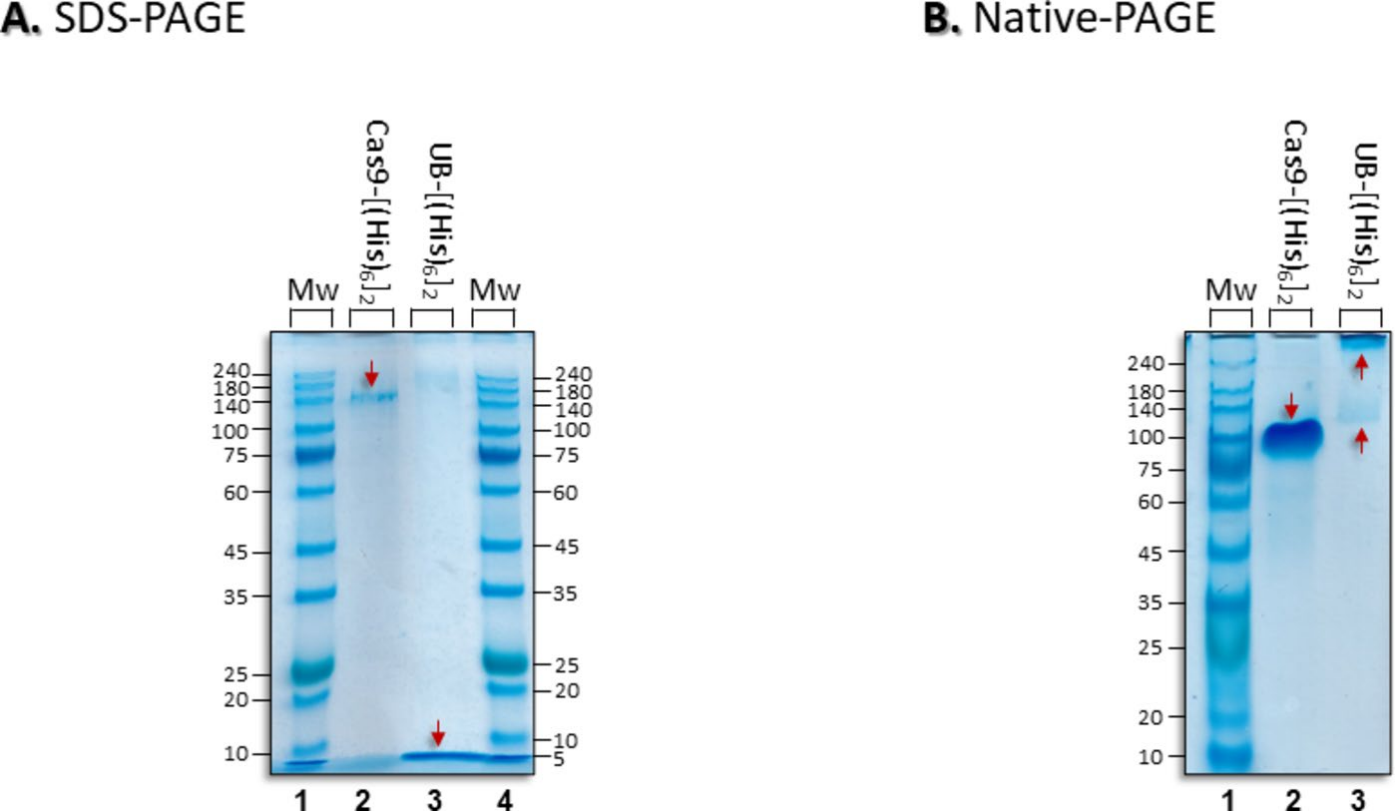
Gel electrophoresis of [UB-His_6_]_2_ and [Cas9-His_6_]_2_: (**A**) SDS-PAGE with reducing agent; (**B**) Native-PAGE. Red arrows indicate protein bands. Gels are Coomassie stained.

SDS-PAGE (Fig. 5A) shows that, following SDS denaturation, both His-tagged proteins migrate on the gel under an electric field at or near the band positions expected for their molecular weights. However, we do find evidence from non-denaturing Native PAGE electrophoresis (Fig. 5B), where mobility is determined by conformation and charge, rather than molecular weight, that, even in the absence of metals, UB-[His_6_]_2_ apparently demonstrates a tendency to aggregate under the native gel conditions.

### Rapid monitoring of solution changes by light microscopy

Light microscopy imaging, performed as described in the “Methods” section, demonstrates the following: (i) Ca^2+^ or Mg^2+^ cations, not known to bind to a His_6_-tag, as do Zn^2+^ and Ni^2+^, are not able to induce large scale precipitation or aggregation of UB-[His_6_]_2_ (Fig. S3A); (ii) None of the four divalent cations, under identical working conditions, lead to protein precipitation when UB-[His_6_]_2_ is replaced by UB-[His_6_]_1_ (Fig. S3B). (iii) Low concentrations of EDTA, histidine or imidazole chelators, capable of competing with the His_6_-tags of UB-[His_6_]_2_ for metal binding, can reverse the process and solubilize the preformed cation-bound UB-[His_6_]_2_ precipitates within 5 min (Fig. S3C).

## Discussion

An oligo-histidine tag is one of the most frequently employed ligands for the purification of recombinant proteins from their expression host cells. Such tags are often found to be compatible with the native structure and proper functioning of the tagged protein and in some cases, their removal may not be required^23^; however, for medicinal applications, the native amino acid sequence alone is generally obligatory^24^. In this communication, we have studied the possibility of using double hexa-histidine ([His_6_]_2_) tagging at the N and C terminal ends of ubiquitin (UB; MW < 10 kDa) and Cas9 (> 160 kDa), both expressed in, and purified from, *E. coli* BL21(DE3) cells, as proof of principle for supramolecular polymerization of proteins *via* [metal: chelator] complexes interaction. Using cryo-TEM imaging, we observe the formation of supramolecular protein fibers or sheets, where the polymer building blocks are folded proteins, but only upon addition of divalent metal cations. That protein secondary structure is preserved is validated by circular dichroism spectroscopy. CD spectra indicated that, following incubation with Ni^2+^ or Zn^2+^, both proteins remain folded and their secondary structure is preserved overall. The minor intensity changes in the spectra upon binding to the cations (Fig. 4) may indicate that the protein experiences small conformational changes following supramolecular polymerization. Ni^2+^ gives rise to fibrous morphology, while the addition of Zn^2+^ primarily results in sheets/membranes. Thus, we are able to demonstrate biopolymerization of UB-[His_6_]_2_ and Cas9-[His_6_]_2_ via [metal: ligand] chelation. Both nano-scale morphologies are obtained following relatively short incubation times at or near room temperature and at physiological pH, thereby opening the possibility of similar biopolymerization of labile proteins that do not tolerate acidic or basic conditions.

Much work remains to be done. Variables which must be optimized include metal identity; metal affinity and coordination number towards the imidazole moieties within the His_6_-tag; working pH; number of histidine residues per His-tag. Rheological measurements should be performed to probe the possibility of gel formation at higher protein and metal concentrations. The particular properties of each protein building block are also likely to determine a unique set of optimal parameters. Poly-ubiquitin, the covalently bound filamentous chain is known to modulate Ub-mediated signaling^25^. Here, we demonstrate a synthetic and potentially dynamically reversible poly-ubiquitin. From the purely structural point of view, polymerization may provide additional benefits. As an example, we may cite NINJ1, a 16-kDa plasma membrane protein, which in its activated filamentous form contributes to cell death^26,27^. Although the low molecular weight of NINJ1 would generally preclude it from being a candidate for cryo-EM structure determination, a cryo-EM structure of NINJ1 in its activated filamentous state, was indeed recently reported^26,27^.

Determining the applicability of ligand/metal based biopolymerization to other water-soluble proteins, as well as the elucidation of the detailed mechanism by which fibers or sheets are generated, are obvious challenges. If these are successfully met, the His-tag approach may allow preparation of a new class of supramolecular biopolymers with important scientific and/or medicinal application.

## Methods

### Materials

NaCl—Sodium chloride, MgCl_2_—Magnesium chloride, Zinc chloride (ZnCl_2_), Nickel chloride (NiCl_2_), Tris, Isopropyl beta-D-1-thiogalactopyranoside (IPTG), Imidazole, phenylmethylsulphonyl fluoride (PMSF), Ethylenediaminetetraacetic acid (EDTA) were all purchased from Sigma-Aldrich, Israel; pET28a (Novagen - Merck, Germany); BL21(DE3) (Thermo Fisher Scientific, USA); lysozyme (Thermo Fisher Scientific, USA); DNase (Invitrogen - Israel).

### Cloning expression and purification of ubiquitin (UB)-[His_6_]_2_

pET28-human ubiquitin plasmid without a tag (from the internal collection of Structural Proteomics Unit (SPU), Weizmann Institute of Science) was used as a template for construction of the double hexa-His tag plasmid. Addition of the double hexa-His tag was performed by Transfer-PCR (TPCR)^28,29^ using 28His_Ubiquitin_F and 28His_Ubiquitin_R primers (Table 1). Sequence analysis was performed to ensure the integrity of the UB-[His_6_]_2_ gene. The final construct pET28-UB-[His_6_]_2_ contains hexa-His-tag and linker at the N-terminal (MGSSHHHHHHSAGSAGSAG) and a linker and hexa-His-tag at the C-terminus (SAGSAGSAGHHHHHH). pET28-UB-[His_6_]_2_ was expressed in LB media in BL21(DE3) cells using Kanamycin (30 µg/ml) as a selection. A 5 L culture was induced at OD_600_ 0.6–0.8, with 200 µM isopropyl beta-D-1-thiogalactopyranoside (IPTG) and grown at 15 °C overnight. The culture was harvested and lysed by a cooled cell disrupter (Constant Systems) in lysis buffer (50 mM Tris pH = 8, 0.5 M NaCl, 20 mM imidazole) containing 200 KU/100 mL lysozyme, 20 µg/mL DNase, 1 mM MgCl_2_, 1mM phenylmethylsulphonyl fluoride (PMSF) and protease inhibitor cocktail. Following clarification of the supernatant by centrifugation, the lysate was applied to a HisTrap-FF_5 mL column (GE Healthcare) and eluted with binding buffer containing 0.5 M imidazole. The eluted Ub-[His_6_]_2_ was injected into a size exclusion (SEC) column (HiLoad_16/60_Superdex75 prep-grade, GE Healthcare) equilibrated with 50 mM Tris pH = 8, 200 mM NaCl, 1 mM EDTA. Pure UB-[His_6_]_2_ migrated as a single peak at 85 mL, was pooled and flash frozen in aliquots using liquid nitrogen and stored at -80 °C.

**Table 1:** Primers used for DNA cloning.

Project	Primer	Sequence (5ʹ to 3ʹ)
Cas9	His_Cas9_F	TCCGCGGGTTCCGCGGGTTCCGCGGGTGACAAGAAGTACTCCATTGGGCTCG
	His_Cas9_R	GTGATGATGATGATGATGGCTGCTGCCCATGGTATATCTCCTTCTTAAAGTTAAACAAAATTATTTC
Ubi4	28His_Ubiquitin_F	GGGCAGCAGCCATCATCATCATCATCACTCCGCGGGTTCCGCGGGTTCCGCGGGTCAGATCTTCGTGAAGACCCTGAC
	28His_Ubiquitin_R	GCAGCCGGATCTTAGTGGTGGTGGTGGTGGTGACCCGCGGAACCCGCGGAACCCGCGGACCCACCTCTGAGACGGAGGACCA

### Cloning expression and purification of Cas9 with double hexa-his tag

pET28-spCas9 (addgene # 47327) with a single C-terminal hexa-His-tag was used as a template for construction of the double hexa-His tag construct. Addition of the N-terminal Hexa-His-tag was performed by inverse-PCR using His_Cas9_F and His_Cas9_R primers (Table 1). The linear PCR product was ligated using the KLD enzyme mix (New England Biolabs, M05544). Sequence analysis was performed to ensure the integrity of the spCas9-[His_6_]_2_ gene. The final construct pET28-spCas9-[His_6_]_2_ contains hexa-His-tag and linker at the N-terminal (MGSSHHHHHHSAGSAGSAG) and a hexa-His-tag and linker at the C-terminus (SRADPKKKRKVAAALEHHHHHH). Expression of pET28-spCas9-[His_6_]_2_ was performed in LB media in BL21(DE3) cells using Kanamycin (30 µg/ml) as a selection. A 5 L culture was induced at OD_600_ 0.6–0.8 with 200 µM IPTG and grown at 15 °C overnight. The culture was harvested and lysed by a cooled cell disrupter (Constant Systems) in lysis buffer (50 mM Tris pH = 8, 0.5 M NaCl, 20 mM imidazole) containing 200 KU/100 mL lysozyme, 20 µg/mL DNase, 1 mM MgCl_2_, 1 mM phenylmethylsulphonyl fluoride (PMSF) and protease inhibitor cocktail. Following clarification of the supernatant by centrifugation, the lysate was applied to a HisTrap-FF_5 mL column (GE Healthcare) and eluted with binding buffer containing 0.5 M imidazole. The eluted Cas9-[His_6_]_2_ was injected into a size exclusion (SEC) column (HiLoad_16/60_Superdex200 prep-grade, Cytivia) equilibrated with 20 mM Tris pH = 8, 200 mM KCl, 10 mM MgCl_2_. Pure Cas9-[His_6_]_2_, migrating as a single peak at 66 mL, was pooled and flash frozen in aliquots using liquid nitrogen and stored at -80 °C. Following thawing, both UB and Cas9 were dialyzed against 30–50 mM Tris, pH 7–7.5 at 8 °C for 24 h prior to all subsequent measurements.

### Cryo-TEM imaging of UB-[His_6_]_2_ and Cas9-[His_6_]_2_

Cryo-TEM samples were prepared in a Leica GP2 plunger (Leica Microsystems, Germany) on lacy carbon grids, pretreated with a 1-min glow discharge in PELCO EasiGlow appliance (Ted Pella, Inc., USA), to render the carbon film more hydrophilic. In the absence of added divalent cations, 5 µL of 3.8 mg/mL UB-[His_6_]_2_ or Cas9-[His_6_]_2_ in 50 mM NaHCO_3_ (pH 6) was applied to the carbon grid in the plunger chamber set to 90% relative humidity, 25 °C. To detect metal/ligand conjugation, 2 µL of 1 mM divalent cations (ZnCl_2_ or NiCl_2_) in 196 µL of 30 mM Tris buffer, pH 7.5, 25 °C were added to 2 µL of 3.8 mg/mL UB-[His_6_]_2_ or Cas9-[His_6_]_2_ in the same buffer, but where the latter contained 100 mM NaCl as well. 4 µL of the mixture was then applied to the grid. Grids were blotted for 5 s using No. 1 Whatman paper and then were plunge-frozen in liquid ethane, cooled by liquid nitrogen to ensure specimen vitrification (fast-freezing). This largely prevents ice crystal formation. Vitrified specimens were stored in liquid nitrogen until viewing in the microscope. For imaging, cryo-specimens were loaded under controlled conditions into a Gatan 626 cryo-holder and kept in the TEM at − 180 °C during the entire procedure. Imaging was performed with a FEG-equipped Talos 200 C TEM (Thermo Fisher Scientific, USA), operated at 200 kV, using the low-dose software of the TEM. Images were recorded by a Falcon III direct-imaging camera using the TIA software package.

### Far UV circular dichroism (CD) spectroscopy

Samples were dissolved at 0.05 mg/mL in double distilled water (DDW) and subjected to CD analysis using a Chirascan CD spectrometer (Applied Photophysics). CD spectra report ellipticity (θ), proportional to the difference in absorbance of left and right circularly polarized light [θ = 3300° (A_L_−A_R_)] as a function of wavelength. A quartz cell of path length 0.1 cm was used for the measurements. The CD spectra were recorded with 2 nm bandwidth resolution in 1 nm steps at 25 °C. CD spectra were corrected for baseline distortion by subtracting a reference spectrum of the corresponding solvent.

### SDS-PAGE electrophoresis

5 µL of 3.8 mg/mL of UB-[His_6_]_2_ or 10 µL of 0.38 mg/mL of Cas9-[His_6_]_2_were loaded onto a 10% polyacrylamide gel, in the presence of SDS and beta mercaptoethanol, prepared according to the protocol of Laemmli^30^. The low molecular weight of UB necessitated increasing the amount of protein run on the gel 10-fold relative to Cas9 in order that the Coomassie staining would provide sufficient contrast.

### Native polyacrylamide gel electrophoresis (native-PAGE)

5 µL of 3.8 mg/mL of UB-[His_6_]_2_ or 10 µL of 0.38 mg/mL of Cas9-[His_6_]_2_were loaded onto a 7.5% native polyacrylamide gel, in the absence of SDS or any reducing agent, prepared according to the protocol of Trudel and Asselin^31^. The low molecular weight of UB necessitated increasing the amount of protein run on the gel 10-fold relative to Cas9 in order that the Coomassie staining would provide sufficient contrast.

### Light microscopy

Hanging drops containing 0.37 mM doubly or singly His_6_-tagged ubiquitin (UB-[His_6_]_2_ or UB-[His_6_]_1_) were incubated with 1 mM divalent cations in 20 mM Tris pH 7.5 in the dark for 1 h at 19 °C. The effect of 2.5 mM EDTA, 5 mM histidine or 5 mM imidazole was evaluated 5 min after chelator addition using the Olympus CX-40 light microscope equipped with an Olympus U-TV1X-2 digital camera.

## Electronic supplementary material

Below is the link to the electronic supplementary material.

Supplementary Material 1

## Data Availability

Data analyzed during the current study will be available upon reasonable request by contacting the corresponding author at guyp@ariel.ac.il.
