# Supplementary material for: His-tag based supramolecular biopolymerization
Source: Sci Rep. 2024 Nov 16;14:28332. doi: 10.1038/s41598-024-78647-1 (PMC11569134; doi:10.1038/s41598-024-78647-1)
Supplement: Supplementary file 1 — Supplementary Material 1 [file 41598_2024_78647_MOESM1_ESM.docx]

**His-Tag based supramolecular biopolymerization**

Mitra Lal ^1^, Ellina Kesselman ^2^, Ellen Wachtel ^3^, Olga Kleinerman ^2^, Yoav Peleg ^4^, Shira Albeck ^4^, Koushik Majhi, ^5^ Mordechai Sheves ^5^ and Guy Patchornik ^1^*****

**Supplementary Information**

^1^ Department of Chemical Sciences, Ariel University, 70400, Israel.

^2^ Department of Materials Science & Engineering, Technion-Israel Institute of

Technology, Haifa 3200003, Israel.

^3^ Faculty of Chemistry, Weizmann Institute of Science, 76100, Rehovot, Israel.

^4^ Center for Structural Proteomics, Weizmann Institute of Science, 7610001, Rehovot,

Israel.

^5^ Department of Molecular Chemistry and Materials Science, Weizmann Institute of

Science, Rehovot 7610001, Israel.

*Corresponding author:

Email: [guyp@ariel.ac.il](mailto:guyp@ariel.ac.il)


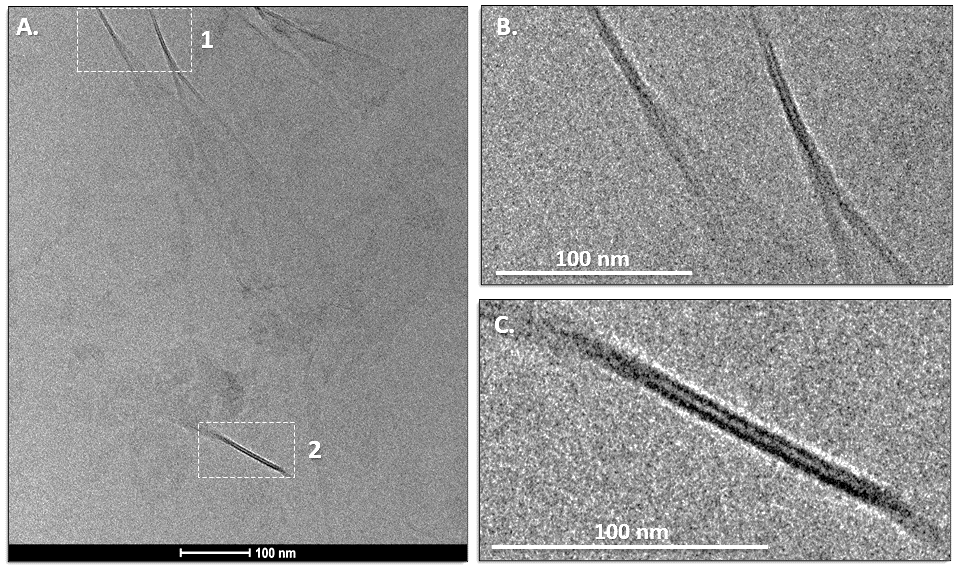


**Figure S1: A.** Cryo-TEM imaging of protein sheets in vitreous ice obtained approx. 10 minutes after addition of 10 µM ZnCl_2_ to 0.05 mg/mL UB-[His_6_]_2_ in 30 mM Tris, pH 7.5, 25 °C. **B-C.** Magnification of dotted white rectangles (1 and 2) in **A,** respectively.


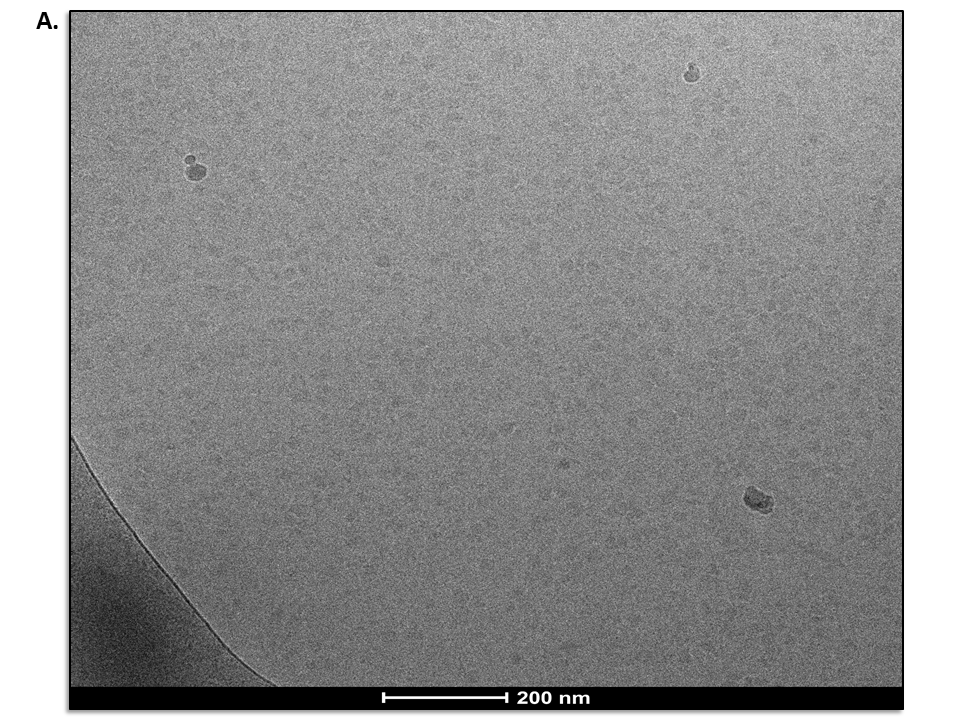

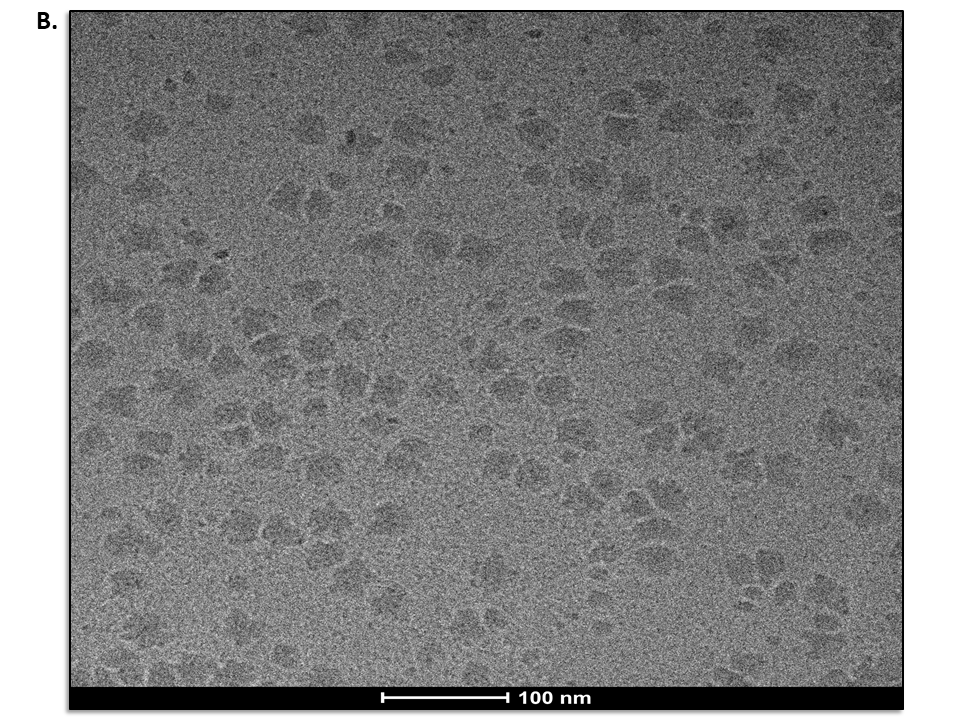


**Figure S2:** **A.** Cryo-TEM imaging of mono His_6_-tagged Ubiquitin UB-[His_6_]_1_ in vitreous ice obtained approx. 10 minutes after addition of 10 µM ZnCl_2_ to 0.05 mg/mL UB-[His_6_]_1_ in 30 mM Tris, pH 7.5, 25 °C. **B**. As in **A,** but at higher magnification.


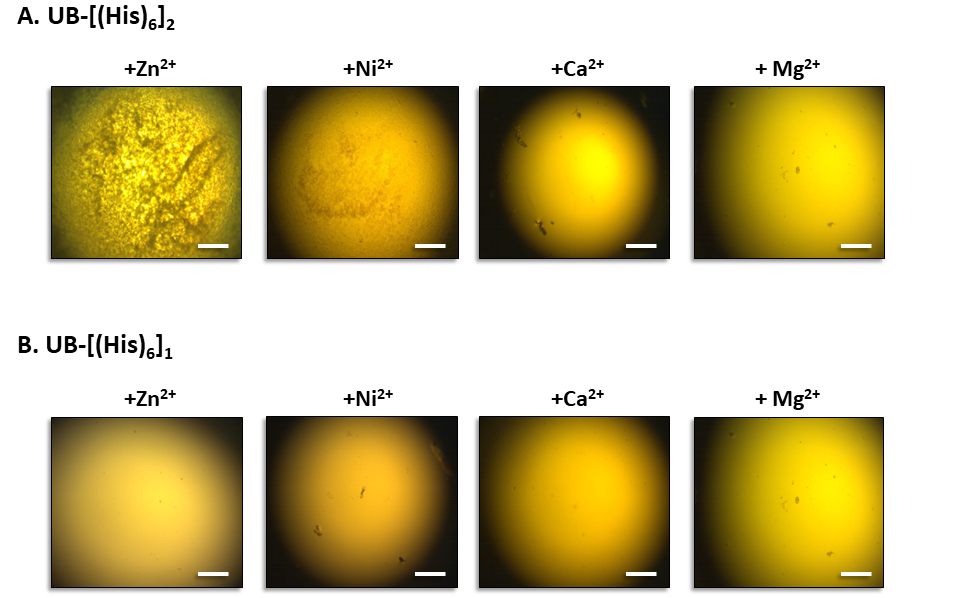


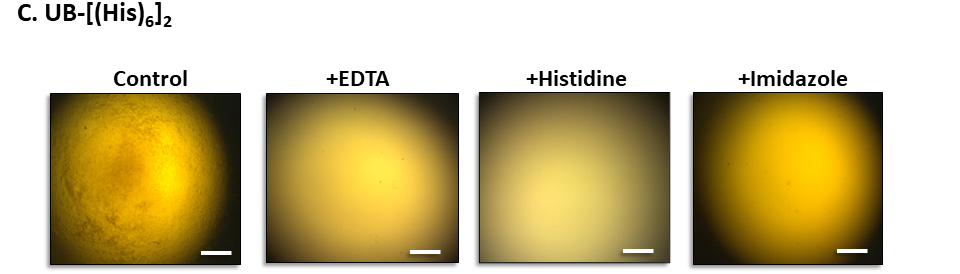


**Figure S3.** Light microscopy images showing the impact of divalent cations on: **A**. Doubly His_6_-tagged ubiquitin (UB-[His_6_]_2_); **B**. Singly His_6_-tagged ubiquitin (UB-[His_6_]_2_). In panels **A** and **B**, protein concentration 0.37 mM, 1 mM metal cations, following 1 hour incubation in the dark at 19^o^C in buffer (20 mM Tris pH 7.5). **C**. Impact of water-soluble chelators on preformed UB-[His_6_]_2_ aggregates. **Control:** UB-[His_6_]_2_ as in panel **A** (+Zn^2+^); **+** (2.5 mM) **EDTA**; + (5 mM) **Histidine**; +(5 mM) **imidazole**. Images in the presence of EDTA, histidine or imidazole were taken 5 minutes after chelator addition. Scale bars represent 200 µm.
